# Strategies for maximizing ATP supply in the microsporidian *Encephalitozoon cuniculi*: direct binding of mitochondria to the parasitophorous vacuole and clustering of the mitochondrial porin VDAC

**DOI:** 10.1111/cmi.12240

**Published:** 2013-12-06

**Authors:** Christian Hacker, Matthew Howell, David Bhella, John Lucocq

**Affiliations:** 1 School of Medicine, University of St AndrewsNorth Haugh, St Andrews, Fife, KF16 9TF, UK; 2 MRC – University of Glasgow Centre for Virus Research8 Church Street, Glasgow, G11 5JR, UK

## Abstract

Microsporidia are obligate intracellular parasites with extremely reduced genomes and a dependence on host-derived ATP. The microsporidium *Encephalitozoon cuniculi* proliferates within a membranous vacuole and we investigated how the ATP supply is optimized at the vacuole–host interface. Using spatial EM quantification (stereology), we found a single layer of mitochondria coating substantial proportions of the parasitophorous vacuole. Mitochondrial binding occurred preferentially over the vegetative ‘meront’ stages of the parasite, which bulged into the cytoplasm, thereby increasing the membrane surface available for mitochondrial interaction. In a broken cell system mitochondrial binding was maintained and was typified by electron dense structures (< 10 nm long) bridging between outer mitochondrial and vacuole membranes. In broken cells mitochondrial binding was sensitive to a range of protease treatments. The function of directly bound mitochondria, as measured by the membrane potential sensitive dye JC-1, was indistinguishable from other mitochondria in the cell although there was a generalized depression of the membrane potential in infected cells. Finally, quantitative immuno-EM revealed that the ATP-delivering mitochondrial porin, VDAC, was concentrated atthe mitochondria-vacuole interaction site. Thus *E. cuniculi* appears to maximize ATP supply by direct binding of mitochondria to the parasitophorous vacuole bringing this organelle within 0.020 microns of the growing vegetative form of the parasite. ATP-delivery is further enhanced by clustering of ATP transporting porins in those regions of the outer mitochondrial membrane lying closest to the parasite.

## Introduction

Microsporidia are intracellular eukaryotic parasites known to cause economically important disease in several animals, including fish, honey bees and silk worms (Desportes-Livage, 2000; Lom and Nielsen, 2003; Cornman *et al*., 2009). They also induce clinically significant disease in immune-suppressed humans, including individuals suffering from AIDS (Didier and Weiss, 2011). Effective and specifically targeted drugs are still scarce (Costa and Weiss, 2000; Didier *et al*., 2005).

More than a thousand species of microsporidia have been described with many more likely to be discovered (Williams and Keeling, 2011). The typical life cycle of microsporidia involves environmental dissemination in the form of a resistant spore, which is able to infect suitable host cells using a unique apparatus termed the polar tube. The polar tube is preassembled in the spore as a coiled organelle that unfurls and acts as a nanosyringe to enter the host cell cytoplasm. The extended tube then functions as a conduit for guiding the sporoplasm into the host cell environment (Wittner and Weiss, 1999; Franzen, 2005). Once in place the parasite proliferates through vegetative (or meront) cell stages and differentiates into sporont, sporoblasts and finally the spore. In many microporidians growth and differentiation take place inside a vacuole which, depending on the species, can be either derived from the host cell or secreted by the microsporidium itself (Hollister *et al*., 1996; Rönnebäumer *et al*., 2008).

Originally thought to be early offshoots in evolution, it became clear that microsporidia have undergone extreme genomic reduction, divesting themselves of metabolic pathways (Weidner *et al*., 1999; Williams and Keeling, 2011) and reducing some organelles such as their mitochondria to tiny relict structures that carry out only a limited array of essential processes (Goldberg *et al*., 2008). Microsporidia are likely to be related to the fungal endoparasite phylum of the cryptomycota (James *et al*., 2013) and are now considered to have undergone a reductive evolution which has resulted in simplified and streamlined adaptations to the intracellular parasitic lifestyle (Wittner and Weiss, 1999).

Most microsporidia appear to have lost the ability to produce ATP in their relict mitochondria and they then rely on the uptake of nutrients and energy substrates from their host (Williams and Keeling, 2003; van der Giezen *et al*., 2005). Accordingly, emerging data have assigned nucleotide transporters to the plasma membrane of the parasite where they could ensure a continuous supply of ATP (Richards *et al*., 2003; Tsaousis *et al*., 2008) which itself can pass through the membrane of the parasitophorous vacuole (PV), known to be permeable to small molecules (< 10 kDa) (Rönnebäumer *et al*., 2008). Interestingly, as the parasite develops from its vegetative/proliferative form into spores, these organisms appear increasingly independent of ATP delivery from the host as they upregulate glycolysis (Heinz *et al*., 2012).

Another way in which microsporidia might influence delivery of nutrients and ATP is by re-organizing the host cell cytoplasm by re-positioning and/or influencing the function of the host cell organelles. Previous studies have suggested that a range of intracellular parasites can influence the organization of host cell cytoplasm in close proximity to the infecting organism (Canning and Hollister, 1992; Sinai *et al*., 1997; Scanlon *et al*., 2004). In *Toxoplasma gondii* one rigorous, and high-resolution, quantitative EM study clearly demonstrated close apposition of mitochondria to the vacuole surrounding the parasites, although a subsequent study failed to show such as relationship (Sinai *et al*., 1997; Magno *et al*., 2005). Here we focused on the host–parasite interaction of *Encephalitozoon cuniculi*, which belongs to a microsporidian genus able to cause human infection (Didier and Weiss, 2006) and harbours one of the smallest eukaryotic genomes found to date (Katinka *et al*., 2001; Keeling, 2001). Previous observations had indicated a clustering of mitochondria around the parasitophorous vacuole of *E. cuniculi* (Scanlon *et al*., 2004), but this study was mainly based on experiments using fluorescent dyes and only qualitative observations at the EM level. It did not investigate the site and mechanism of interaction in detail. Here we report a rigorous quantitative assessment of host cell mitochondria, in cells infected with *E. cuniculi*.

Taken together, our results reveal that host cell mitochondria bind directly to the parasitophorous vacuole membrane via electron dense bridging-structures (< 10 nm length). The mitochondria bind preferentially to the early vegetative ‘meront’ cell stages that are themselves only found at the vacuole periphery and bulge into the host cell cytoplasm. In a broken cell system we show that this interaction is retained after removing soluble cytosolic factors but in the same system is sensitive to digestion by proteases. By using a fluorescent dye that reports on mitochondrial membrane potential, we find the function of adherent mitochondria is indistinguishable from mitochondria elsewhere in the cell. Interestingly, we also find that VDAC molecules, the ATP exporting voltage gated channels of the mitochondrial outer membrane, become concentrated at the sites of mitochondrial binding. We conclude that *E. cuniculi* induces direct protein-dependent binding of host mitochondria to the parasitophorous vacuole surrounding the vegetative meront cell stages. This binding is combined with mitochondrial ATP-delivering channels at the vacuole membrane to facilitate delivery of ATP from host cytoplasm to the growing and differentiating parasite.

## Results/discussion

### Quantification reveals a single layer of mitochondria at the PVM

**Figure 1:**
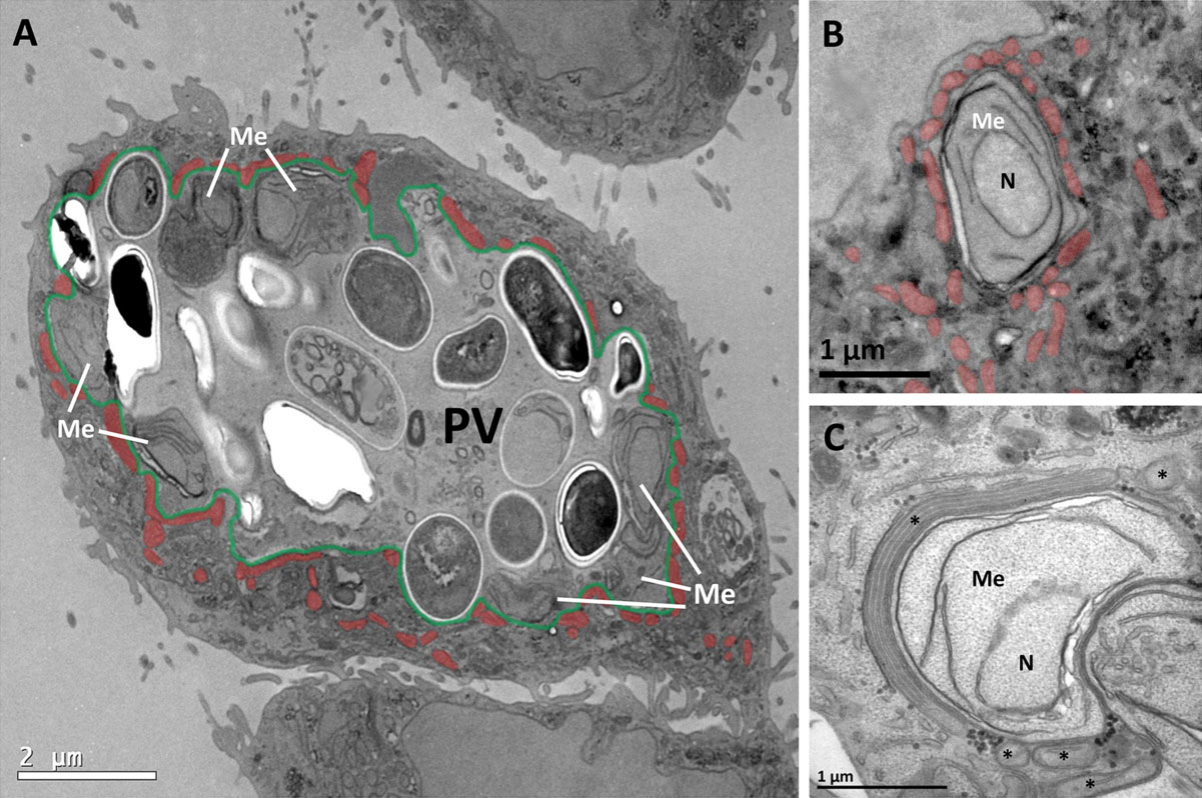
Host cell mitochondria associate with the parasitophorous vacuole of *E. cuniculi*.A. Overview of a RK-13 cell infected with a large *E. cuniculi* PV. The PV (false-coloured in green) encloses a range of different microsporidial stages including meronts and spores (Me, meronts; remaining cells inside PV are spores, recognizable by their discernible cell wall and the ovoid cell shape). Meronts are exclusively located close to the PVM whereas spores differentiate within the body of the PV lumen. Numerous mitochondria (false-coloured in orange) are clustered at the PVM surface preferentially next to meronts.B. Profile of an early meront stage with mitochondria (false-coloured in orange) that ‘cover’ the PVM over large proportions of meront surface.C. Higher magnification of a PV region with a meront bulging into the host cytoplasm. The meront is extensively covered with a mitochondrion that is attached over the full length of its profile. Notice the consistent and close intermembrane spacing between outer mitochondrial membrane and PVM.Me, meront; asterisk, mitochondrion; N, nucleus of meront.

The highly simplified relict mitochondria of microsporidia have lost the ability to produce ATP (Tsaousis *et al*., 2008; Williams *et al*., 2008), and so these organisms must have evolved different strategies of sustaining their energy requirements. We tested the hypothesis that the microsporidian *E. cuniculi* can modulate its interaction with the host to facilitate ATP delivery. Using conventional electron microscopy we first investigated the nature and extent of the re-organization of the host cell cytoplasm close to the PV. The membrane of the PV (PVM) can be distinguished easily and careful study of *E. cuniculi*-infected RK-13 cells revealed apparent clustering of mitochondria around the PVM (Fig. 1) as described in previous reports (Scanlon *et al*., 2004). A closer examination of the host cell mitochondria in *E. cuniculi*-infected cells revealed that mitochondria appear to be closely applied to the PV (Fig. 1A–C). In order distinguish between clustering/aggregation and direct binding in the region of the PV we used spatial stereology, which maps the quantities of organelles in the cytoplasm with minimal bias (McCullough and Lucocq, 2005). In the case of clustering, the density of mitochondria would fall off gradually away from the vacuole. In the case of direct binding an abrupt fall in mitochondrial density would be observed.

**Figure 2:**
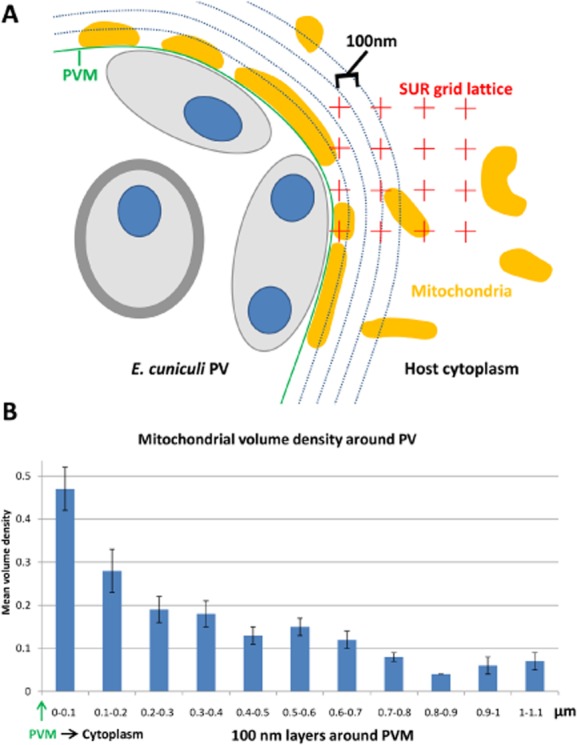
Gradient of mitochondrial volume density around the PVM. Volume density was estimated using point counting and surface fraction by intersection counts on micrographs containing regions of *E. cuniculi* PVs (see *Experimental procedures*).A. Schematic depiction of 100 nm layers surrounding the PVM (only 4 layers shown) used for the volume density estimates of mitochondria in the experiment. The SUR grid lattice is represented illustratively and would extent over the whole area of the micrographs.B. Estimated mean volume density of mitochondria within 100-nm-wide shells around the PV. The highest volume density of mitochondria could be detected in the first 100 nm layer around the PV (0.47, SEM 0.05) followed by a 40% step-wise decrease in volume density in the second 100 nm layer (0.28, SEM 0.05) indicating that mitochondria form a single layer of profiles around the PVM by direct binding rather than mere clustering. Error bars indicate the SEM.

We found an aggregate mean volume density of mitochondria for all the sampled cytoplasm of 0.18 [*n* = 1164, standard error of the mean (SEM) = 0.01], which indicates that 18% of the host cell cytoplasm in the general region of the PV was occupied by mitochondria. However, when the volume density was estimated in concentric 100 nm shells of cytoplasm around the PV (Fig. 2A), there was a clear pattern of mitochondrial concentration close to the PV (Fig. 2B). Across the first three 100 nm bands, the volume density decreased from 0.47 (SEM 0.05), to 0.28 (SEM 0.05) and 0.19 (SEM 0.03) respectively. This represented a drop of 40% between 0–100 and 100–200 nm and 20% between 100–200 and 200–300 nm. To better understand the observed changes of mitochondrial density around the PV we used a relative index (RI). This compared the estimate for each layer with the aggregate value for all layers as a ratio and Chi-squared tests were then used to statistically evaluate the distribution of point counts throughout the layers. The RI fell from 2.54 to 1.47 to 1.00 respectively and was less than 1.00 for all layers beyond 200–300 nm [for all layers the Chi-square was 377.35 (*P *<* *0.001; df 10)]. The partial Chi-square for the first 100 nm was 237.99 (63% of total) reflecting the vast majority of mitochondrial volume was located in this layer directly covering the PVM. We concluded that the major accumulation of mitochondria occurred within 200 nm of the PVM.

To assess how many layers of mitochondria might contribute to the observed distribution we measured the mean calliper diameter of mitochondrial profiles in a direction orthogonal to their long axis. The mean calliper width was 150 nm (range 100–222 nm, SEM 3.85 nm; *n* = 59) indicating that the dramatic fall in density across 200 nm represents a single layer of mitochondria.

### Close, consistent and uniform association of PVM with host cell mitochondria

On further examination we found an extremely close association between the outer mitochondrial membrane (OMM) and the PVM with both membranes being in close association over the full length of the mitochondrial profile (Fig. 1C). The average distance between the OMM and the PVM was 7.7 nm (median 6.45 nm) with a standard error of the mean of 0.51 nm (*n* = 68) with 68% of all measurements within 2.24 to 8.24 nm. At irregular intervals the interface between mitochondria attached to the PVM appeared distorted with visible deformations extending from the OMM to the plasma membrane of the parasite (Fig. S1). Close inspection of PVM regions showed that mitochondrial profiles often matched the shape of the PV closely, even protruding into the PV lumen to follow invaginations of the PV (see Fig. 1A and C).

**Figure 3:**
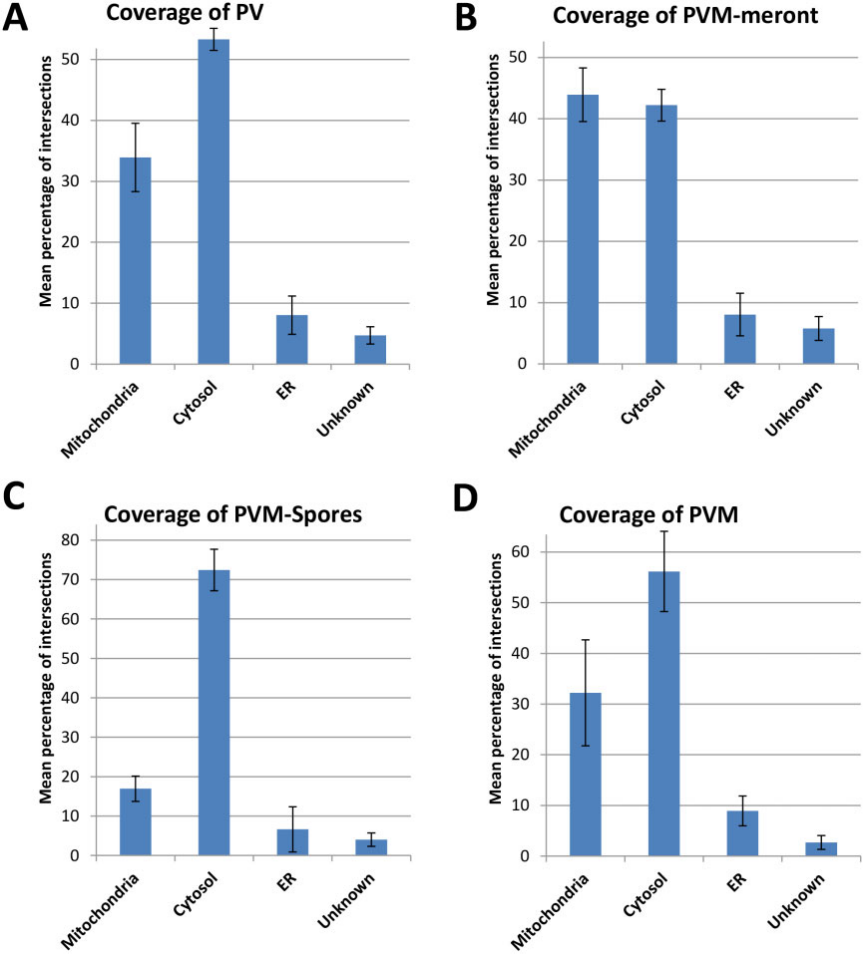
Fraction of PVM surface covered with host cell organelles.A. Fraction of the total PVM membrane surface in direct contact with host organelles.B–D. The fraction of the PVM membrane surface in contact with host organelles in regions of PVM overlaying different microsporidian stages (or lack of them). The mean fraction of the PVM occupied by mitochondria was significantly higher for PVM covering meronts compared PVM associated with spores indicating a preferential binding of mitochondria to early vegetative cell stages.Error bars indicate the SEM.

Emerging data suggest that the early vegetative stages of microsporidians might be more dependent on host cell ATP than the spore stages because as they differentiate they become walled off from the outside world by a thick resistant cell wall. This may explain why the microsporidian *Trachipleistophora hominis* is known to upregulate their glycolytic pathway as spores differentiate (Heinz *et al*., 2012). We therefore investigated whether the association of mitochondria was specific for the underlying cell stage of *E. cuniculi*. This was done by estimating the fraction of the PVM surface occupied by mitochondria using line intersection counting (see *Experimental procedures*). This analysis revealed that overall 34% of the total PV surface was covered with mitochondria that were applied closer than 8 nm to the PV membrane (Fig. 3A, mean value, *n* = 3, 185 to 256 intersection counts per experiment). By comparison the proportion of the host cell plasma membrane and host nucleus covered by mitochondria analysed was 0 (single experiment; 148 intersections) and 0.1 (single experiment; 31 intersections) respectively. Next we analysed subregions of PVM related to meronts, spores and empty portions of the PVM (Fig. 3B–D). The mean fraction of the PVM occupied by mitochondria was 0.44, 0.17 and 0.32 (*n* = 3; intersections as described for Fig. 3A) for meronts, spores and empty portions of the PVM respectively. Thus the mitochondria attach primarily in proximity to the vegetative cell stages of the parasite. Indeed we observed that early meront profiles that appeared isolated in the cytoplasm (likely soon after infection) were often completely surrounded by mitochondria (Fig. 1B). Significantly there was a dramatic drop between the coverage of PVM over meronts compared to spores, which indicated that as spores develop the diffusional distance for ATP increases. To investigate this effect quantitatively we measured the apparent distance between the parasite plasma membrane (the site of ATP transporters) and the nearest mitochondrial profile. This distance increased from 17.6 nm (SEM 6.39 nm) to 329.2 nm (SEM 74.26 nm) for meronts and spores respectively (*n* = 40 meronts and 40 spores); so the diffusional distance for ATP for meronts averaged less than 0.020 microns which is an order of magnitude nearer than for spores.

Our data were consistent with a direct binding of mitochondria to the PVM overlying meront stages of the parasite and so we looked for further adaptions that would increase the surface of mitochondria associated with the meront-PV. Using stereology we discovered that almost 2/3 (65.78%, 226–368 intersections per experiment; Fig. S2) of the meront is in contact with the PVM surrounded by the host cytoplasm, which indicates that the meront also bulges from the PV into the host cell cytoplasm (see Fig. 1C). Thus a significant portion of the meront PVM surface is in direct contact with host cell mitochondria, which predominantly organizes at the early vegetative meront cell stages of the parasite in a tight association by forming a single layer of mitochondria to cover almost half of the fraction of the meront plasma membrane that is in contact with the cytoplasm.

To evaluate whether the intimate association of PVM and host mitochondria was present along the whole length of each individual mitochondrion we used electron tomography. Two hundred and fifty-nanometre-thick sections of *E. cuniculi*-infected RK-13 cells were prepared and tilt series made at locations where mitochondria were in direct contact with the PVM. The extent of this connection was then analysed by tracing the OMM associated to the PVM throughout the reconstructed tomograms. The interaction of both membranes thereby appeared seamless over the full length of the analysed tilt series (Fig. S3), indicating that the mitochondria associated with the PVM are closely attached along the entirety of their organelle profile. Corresponding observations were obtained by analysing consecutive serial ultra-thin sections. We found that 40.6% of the total outer membrane surface of attached mitochondria is in direct contact with the PVM (*n* = 10 complete mitochondria, SEM = 1.41%; see *Experimental procedures*). Therefore this percentage corresponds to almost all of the mitochondrial outer membrane surface facing the vacuole, and we conclude that mitochondria are bound to the PV along their entire length. As described quantitatively in Fig. 3A–D we observed an association between stretches of endoplasmic reticulum (ER) and the PVM with ER cisternae closely following the curvature of the PVM (Fig. S4). However in contrast to the mitochondrial attachment the ER was not continuously in direct contact with the PVM and was relatively much more minor in extent.

### No PVM host cell organelle interaction in *T. hominis*-infected RK cells

One explanation for clustering of organelles in microsporidia-infected cells is crowding of organelles in the peripheral cytoplasm of the host as the PV enlarges. Previously this effect has been ruled out in studies of organelle interactions with vacuoles of other intracellular parasites *Coxiella burnettii* and *Leishmania amazonenzis* (Sinai *et al*., 1997). RK-13 cells were infected with *T. hominis*, which like *E. cuniculi,*is a human pathogen isolated from AIDS patients (Hollister *et al*., 1996). As a microsporidian and obligate parasite, *T. hominis* also relies on the uptake of ATP from its host (Heinz *et al*., 2012). Interestingly, the examination of *T. hominis* PVs presented a very different morphology in comparison with the results obtained in the same cell type infected with *E. cuniculi*. We could not observe any obvious clustering of mitochondria around the PVM of *T. hominis*. Qualitative analysis showed that mitochondria were distributed throughout the cytoplasm without any apparent direct contact of OMM with the PVM (Fig. S5) and stereology showed that the surface fraction of the PVM occupied by mitochondria on the recorded micrographs of *T. hominis* PVs was 0.0289 (510 intersection counts on 21 analysed PVs) verifying that *T. hominis* does not cluster host cell organelles around their PVM. We conclude that the mitochondrial binding is a specialization in *E. cuniculi*. This likely reflects the diverse origin of PVM – the vacuole in *E. cuniculi* is host cell derived while the vacuole of *T. hominis* appears to originate from the parasite itself (Hollister *et al*., 1996; Rönnebäumer *et al*., 2008).

### Binding of host cell mitochondria to the PVM in broken cell system

**Figure 4:**
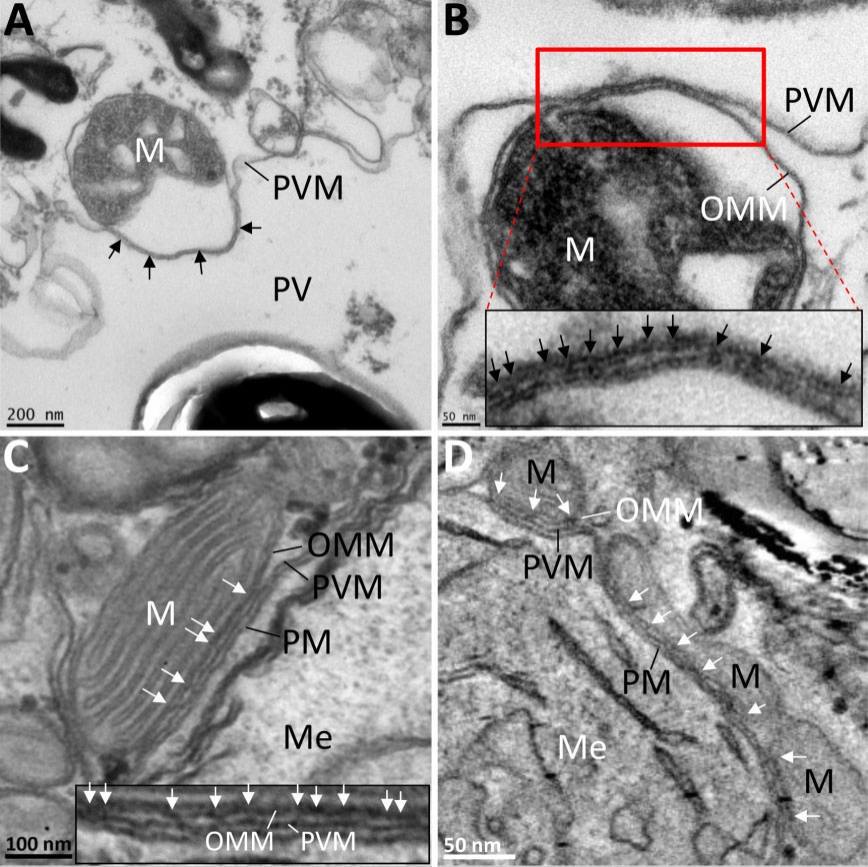
Binding of host cell mitochondria to the PVM in a broken cell system reveals electron dense bridges.A. Region of an *E. cuniculi* PV in a broken RK-13 cell. The broken cell appears devoid of granular cytosol and parasite cells as well as host organelles are identifiable. A mitochondrial profile can be seen bound to the PVM (black arrows). This interaction remains intact even when discernible separation of the outer mitochondrial membrane inner membrane has occurred.B. Mitochondrial profile bound to the PVM. Several electron dense bridges (better seen in the higher magnification view) are visible, stretching between the outer mitochondrial membrane and the PVM.C. Mitochondrial binding to the PVM in an intact cell. Close observation of the intermembrane space between the PVM and the OMM reveals electron dense linkages (see white arrows in micrograph and enlargement).D. Snapshot of an electron tomogram showing several mitochondria bound to the PVM surrounding a meront cell. Electron dense bridges can be seen spanning the OMM and PVM (see white arrows).M, mitochondrion; Me, meront; OMM, outer mitochondrial membrane; PV, parasitophorous vacuole; PVM, parasitophorous vacuole membrane.

We next investigated the nature of the close interaction between host cell mitochondria and the PVM. Broken cells are a powerful way to investigate the nature and mechanisms of organelle interactions (Beckers *et al*., 1987). This experimental system allows access to the cytoplasmic aspect of endomembranes such as Golgi, endosomes, nuclear envelope and mitochondria. In order to evaluate the stability of the association of mitochondria with the PVM, we broke the host cells by scraping, a procedure which releases putative soluble factors involved in maintaining the association of mitochondrial binding to the PV. Broken *E. cuniculi*-infected RK cells were incubated on ice for 1 h before embedding and sectioning for electron microscopy. After this procedure broken cells showed clear evidence of release of cytosol, as determined by discernible reduction of granularity and electron density (although residual cytoskeletal components were observed) and swelling and disruption of a number of organelles. In such cells we found that host cell mitochondria were still associated with the PVM and crucially, the binding of mitochondria to the PV was still preserved with the OMM remaining attached to the PVM even when the main body of the organelle was grossly swollen or even lost (Fig. 4A and B).

We next quantified the effect of breaking the cells on the mitochondrial binding stereologically by comparing line intersection counts on sampled micrographs of PVs from broken and intact cells present in the same preparations (see *Experimental procedures*). We found that in permeabilized cells 25% of the PVM surface was covered by mitochondria, while in unbroken cells in the same ultrathin sections, 24% of the PVM surface fraction was associated with mitochondria (*n* = 24 broken cells, 387 intersection counts; *n* = 17 intact cells, 266 intersection counts respectively). We conclude that breaking the host cells did not alter the PVM – mitochondrial interaction indicating a strong and direct attachment independent of the presence of cytosol.

The close proximity and stability of the PVM – mitochondria association suggested that there were direct connections between the membranes involved; and so we took advantage of the enhanced contrast of cytoplasmic structures evident in permeabilized cells (Smythe *et al*., 1989). Indeed we could visualize striking examples of electron densities between the PVM and the OMM using this method. The putative linkages now appeared as discrete bridges distributed irregularly across the length of the mitochondrial – PVM contact zone (Fig. 4B). In some cases we could observe striations with a consistent periodicity resembling zipper-like connections grouped into clusters, although there were large stretches of membrane contacts containing no visible linkages. The mean length of those connections was 6.52 nm (SEM 0.38 nm, *n* = 71), closely fitting the length distribution between the PVM and the OMM we measured in intact cells (7.7 nm; see above and Fig. S6). Electron dense bridges were also observed in intact cells (Fig. 4C) as well as in electron tomograms processed from tilt series of 250 nm thick sections of the same material (Fig. 4D and Fig. S3). As expected detection of the cross bridges in thawed frozen sections prepared according to the Tokuyasu method (see *Experimental procedures*) proved more difficult – by this method periodic structures such as clathrin coated pits are particularly difficult to visualize.

### Protease treatment of structural PVM – mitochondrial linkages

**Figure 5:**
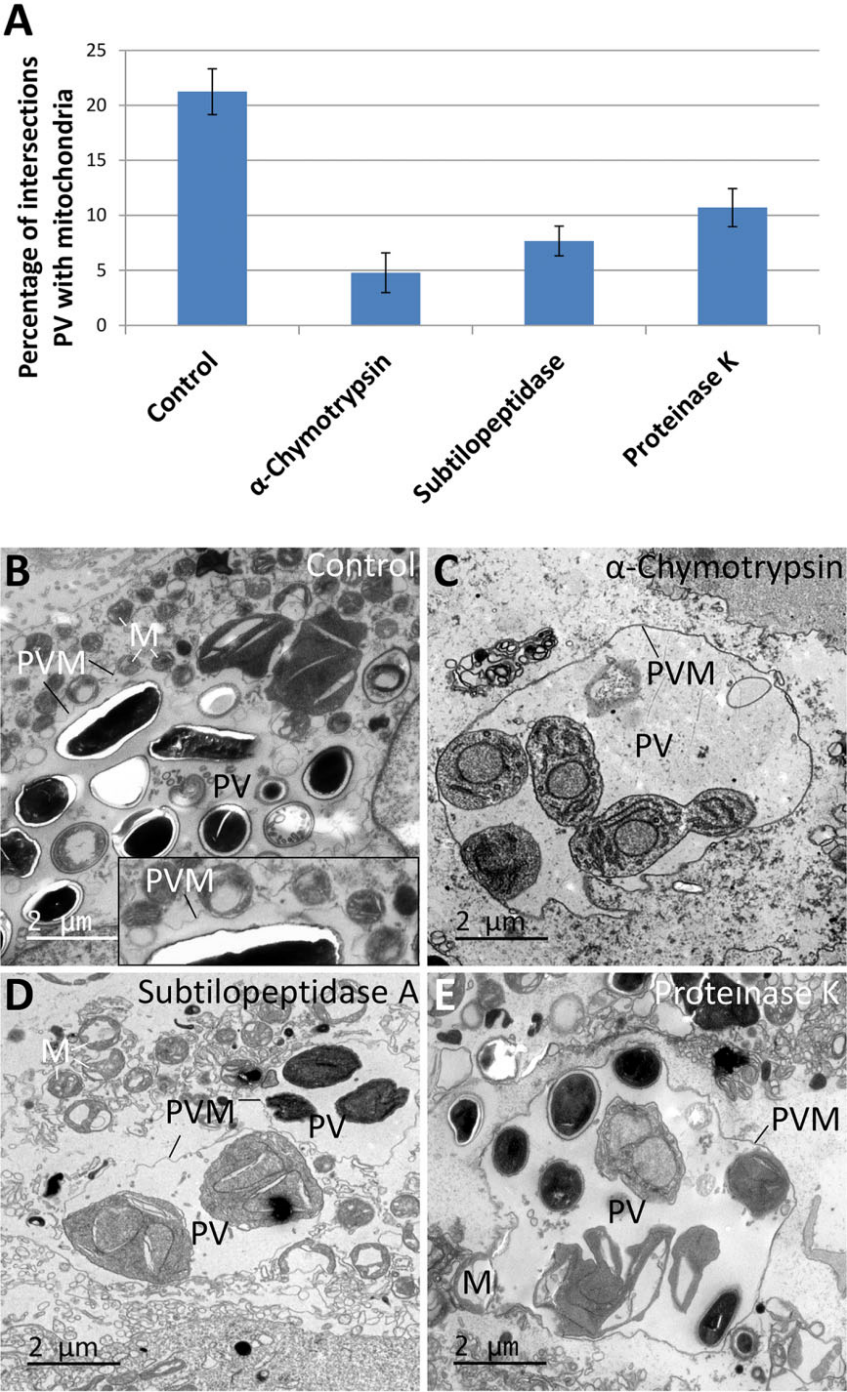
Protease-sensitive binding of mitochondria to the PVM in broken cells.A. Estimates of mean PVM surface fraction occupied by mitochondria in broken cells after protease treatment. Error bars indicate the SEM.B. Binding of mitochondria to the PVM in broken cells without protease treatment as a control. Several mitochondrial profiles can be seen bound to the PVM (see enlargement).C–E. Show reduced mitochondrial binding to the PVM in broken cells after protease treatment.M, mitochondrion; PV, parasitophorous vacuole; PVM, parasitophorous vacuole membrane.

Our next aim was to evaluate whether the binding between PVM and OMM is dependent on proteins. To do this we treated the permeabilized cells with a range of different proteases and quantified the fraction of PVM surface occupied by mitochondria with intersection counting. Broken *E. cuniculi*-infected RK cells were treated with 0.1 mg ml^−1^ of either proteinase K, subtilopeptidase A or α-chymotrypsin for 1 h on ice. This led to an apparent separation of PVM and mitochondria with the PVM appearing to be largely devoid of host organelles or electron dense bridges (Fig. 5C–E); while in intact cells there was no change in the association of mitochondria PVM (Fig. 5B). Quantification confirmed the marked reduction in mitochondrial binding to the PVM in protease-treated cells (Fig. 5A). In untreated broken cells, the PVM surface fraction occupied by mitochondria was 21.24% (*n* = 12–24 PVs in three individual experiments, 157–387 intersections per experiment, SEM 2.1%) and this was reduced to 4.79%, 7.66% and 10.69% after treatment with α-chymotrypsin, subtilopeptidase A and proteinase K respectively. We conclude that mitochondria and PVM are joined together in a protein-dependent manner most likely by means of the electron linkages already described. Further studies are under way to investigate the partners involved in this interaction.

In summary we found electron dense structures bridging between PVM and mitochondria and found the interaction is sensitive to proteases strongly suggesting the interaction is dependent on protein–protein interactions. These data explain why the mitochondria are bound in one layer. We speculate that this interaction was collectively of high affinity, thereby explaining the deformation of the PVM to cover significant proportion of the vegetative early forms of the parasite (see Fig. S1). A direct binding mechanism is consistent with a previous study showing that clustering of mitochondria around the PV was insensitive to depolymerization of microtubules using the drugs albendazole or demecolcine in *E. cuniculi*-infected cells (Scanlon *et al*., 2004).

### Spatial analysis of membrane potential in host cell mitochondria in *E. cuniculi*-infected cells

**Figure 6:**
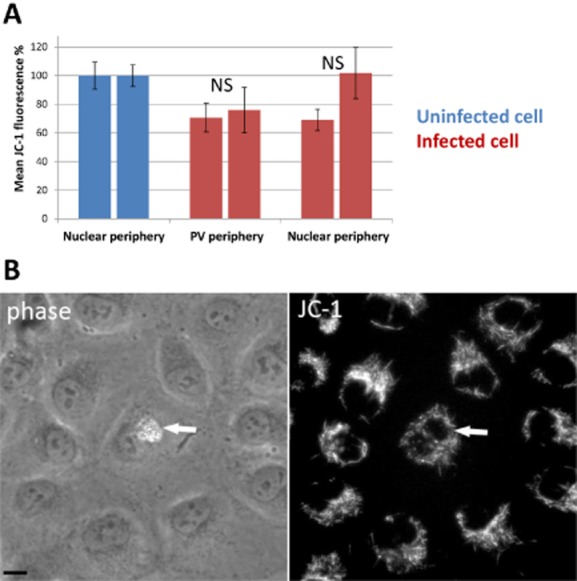
*In situ* assessment of mitochondrial membrane potential in *E. cuniculi*-infected RK cells using the membrane-permeable dye JC-1.A. Quantification of the fluorescent intensity of JC-1 indicating levels of mitochondrial membrane potential in uninfected compared to infected cells from two individual experiments. The fluorescent intensities were normalized in relation to the mean level of JC-1 fluorescence in uninfected cells. Measurements were made adjacent to either the nucleus or PV (see *Experimental procedures*). The global membrane potential is decreased by about 20% in infected cells. The mitochondrial activity was comparable in regions adjacent to the PV compared to locations adjacent the nucleus, indicating that *E. cuniculi* has no direct influence on the local levels of ATP production. Error bars indicate the SEM. The results for non-infected cells (*n* = 19) compared to juxta-PV locations (*n* = 12) or juxta-nuclear locations (*n* = 12) in infected cells were not statistically significant (NS; *P* values > 0.05 using the Mann–Whitney test).B. JC-1 fluorescence in RK-13 cells. The white arrow highlights a cell infected with *E. cuniculi*. A PV containing numerous cell stages of the parasite can be seen. The fluorescence of JC-1 appears evenly distributed in the infected cell. Note the higher intensity of JC-1 in uninfected cells in the same frame. Phase = phase contrast; bar 10 μm.

The observed close spatial relationship mediated by direct binding of PVM and host cell mitochondria is a powerful mechanism for enhancing delivery of ATP to the early stages of the parasite, but this binding could also modulate (and possibly accelerate) the function of the bound mitochondria. To test this idea we measured mitochondrial activity inside of living cells using membrane-permeable dye JC-1 (reviewed by Perry *et al*., 2011). JC-1 responds to the membrane potential of mitochondria in cells infected with *E. cuniculi* emitting fluorescence depending on the mitochondrial membrane potential. Highly active ATP-production is indicated by the accumulation of red fluorescent J-aggregates (Smiley *et al*., 1991). We compared the red JC-1 signal of mitochondria close to the PVM with that of mitochondria located elsewhere in the same infected cells (this comparison effectively controls for any changes in cell permeability related to the infection process). RK-13 cells infected with *E. cuniculi* at various cell stages were therefore stained with JC-1 and the fluorescent intensity measured in living cells in two different locations per cell profile (close to the PVM and close to the nucleus, see *Experimental procedures*). In these regions we also estimated the volume density of mitochondria using EM (see *Experimental procedures*) at locations similar to those used for JC-1 analysis. We found that there was no marked difference in the volume density of mitochondria in the regions used for the JC-1 measurements in infected cells (0.21 for mitochondria adjacent to PV, 0.19 adjacent to nucleus). This was an important control because immunofluorescence intensity is derived from volumes of cytoplasm in the sampled regions that may vary in their concentration of mitochondria. The results show that in infected cells the levels of fluorescent intensity from JC-1 at the PV and nucleus was comparable (Fig. 6) suggesting that the parasite does not influence the activity of adherent mitochondria and their production of ATP.

Interestingly we could detect a 20% decrease in fluorescent intensity in the mitochondria of infected compared to non-infected cells. This might reflect a general reduction of mitochondrial activity due to the presence of the parasite perhaps as a prelude to the mitochondrial depolarization that occurs in the early stages of apoptosis (Mancini *et al*., 1997). In this case the adherence of mitochondria could be an adaptation to maintain a supply of ATP in a background of failing mitochondria in dying cells (Tsaousis *et al*., 2008). To test the idea we plotted the recorded JC-1 intensities of mitochondria in close contact with the PVM against the size of the corresponding PV. However we found no evidence for a correlation between the growth of the PV and a reduction in mitochondrial activity (data not shown).

Taken together the analysis of the mitochondrial membrane potential in *E. cuniculi*-infected RK cells revealed that the parasite appears not to actively regulate the capacity of mitochondria to produce ATP in the region close to the PV. Rather unexpectedly the parasite appears to compromise mitochondrial function in the whole cell en masse. Thus concentrating mitochondria close to the PVM is a mechanism for optimizing the ATP delivery in a likely background of a failing energy supply.

### Localization of VDAC in mitochondria bound to the PVM

**Figure 7:**
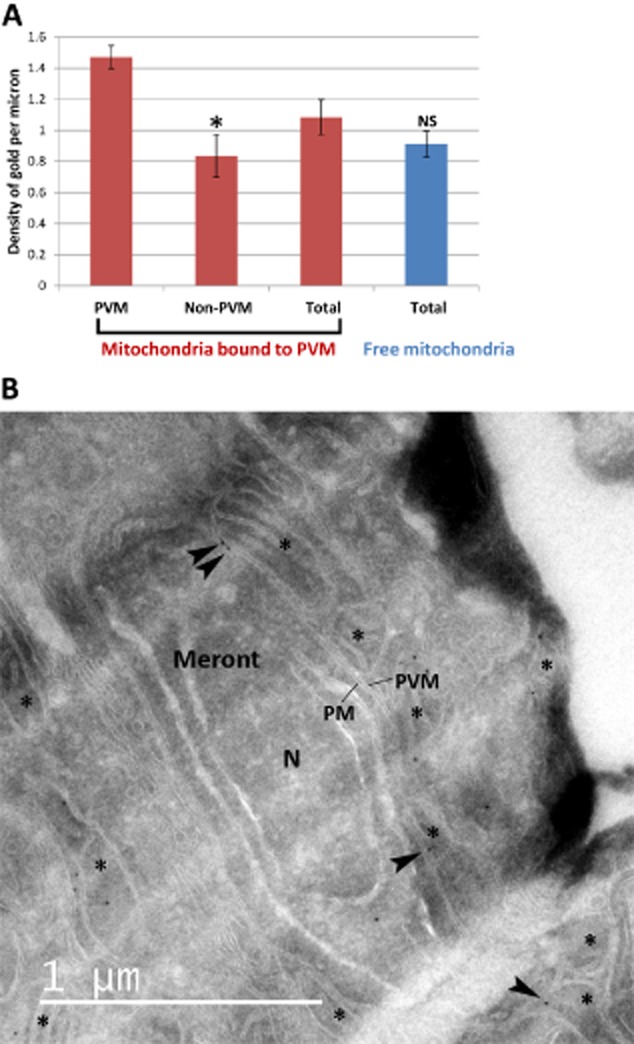
Quantification of the density of VDAC in mitochondria bound to the PV in thawed cryo-sections of *E. cuniculi*-infected RK-13 cells. Cryo-sections were labelled with an antibody against the mitochondrial porin, VDAC. Micrographs were taken at regions containing the PVM.A. Mean densities of VDAC gold labelling from five individual experiments. The membrane boundary lengths of interest were estimated using intersection counting (see *Experimental procedures*) and counts of 10 nm protein-A gold made. As a control the VDAC signals in the endoplasmic reticulum and the plasma membrane were also estimated in three initial experiments producing an estimated gold particle density of 0.0205 (138 to 179 intersection counts; SEM 0.0046) and 0.0156 (46 to 68 intersection counts; SEM 0.0081) for the ER and the PM respectively. No gold particles were detected over profiles of the Golgi apparatus and over the plasma membrane of the parasites. Error bars indicate the SEM. Labelling density over juxta-PV membranes was significantly higher (*) than labelling density over PV-free membranes belonging to the same mitochondria (Mann–Whitey test, *P* ≤ 0.01, *n* = 5 in each case). When the ‘global’ gold labelling density over PV-bound mitochondria was compared to that over mitochondria free in the cytosol the difference was not significant (NS) using the Mann–Whitney test (*P* > 0.05; *n* = 5 in each case).B. Micrograph representative of the quantitative data displaying a meront with several mitochondria bound to the PVM. VDAC positively labelled with gold particles at the PVM–OMM interaction site is highlighted with black arrowheads. Asterisks, mitochondria; N, nucleus of meront; PM, plasma membrane of meront; PVM, parasitophorous vacuole membrane.

Finally we investigated whether *E. cuniculi* could potentially enhance the ATP supply by influencing the machinery involved in mitochondrial ATP translocation. ATP, ADP, Ca^2+^ and other metabolites are channelled through the outer mitochondrial membrane into the surrounding cytosol by specific porins, primarily by the voltage dependant anion-selective channel (VDAC; for a review see Colombini, 2012). One possibility for enhancing ATP delivery would be an increased concentration of VDAC channels at the outer mitochondrial membrane facing the PVM. To test this idea we labelled thawed cryo-sections of *E. cuniculi*-infected RK cells with an antibody against VDAC and quantified the density of gold particles in mitochondria surrounding the PV (see *Experimental procedures*). The antibody labelling was found almost exclusively over outer mitochondrial membranes and the density of VDAC labelling was 1.76-fold higher at PVM-mitochondrial interaction sites compared to the rest of the OMM of the same mitochondrial profiles (Fig. 7). The labelling was also 1.61-fold higher along the PVM-mitochondria interaction compared to the labelling over non-bound mitochondria. Importantly these results are consistent with a redistribution rather than a change in labelling efficiency because (1) the overall labelling density on the PVM-bound mitochondria was not markedly different from overall labelling over the non-bound mitochondria, and (2) the distribution of labelling across the outer membrane was not significantly different (for example 40.59% of gold particles at the PVM–OMM interaction site in bound mitochondria compared to 32.22% of gold particles in the OMM of free mitochondria; Chi-squared value 3.83; degree of freedom = 2; *P* > 0.1; data that speak against domain-specific changes in conformation that might drive increases in antibody binding).

There is a precedent for spatially directed function of VDAC because it is known to bind cytosolic ATP-dependent enzymes such as hexokinase I and II (Wilson, 1995; Pedersen *et al*., 2002). This binding is thought to channel ATP into the glycolytic pathway and a similar interaction could do the same at the *E. cuniculi* PVM. In this case VDAC could bind directly to proteins in the PVM providing a structural and functional link between mitochondrion and meront and further experiments are underway to analyse the functionality of VDAC in relation to ATP-delivery directly to the parasite as well as identifying binding partners of VDAC at the PVM–OMM interaction site. The direct proof that VDAC channels ATP into *E. cuniculi* will be extremely challenging but experiments that inhibit the functionality of VDAC – potentially influencing the growth and proliferation of the parasite – will be informative. Similarly, the identification of VDAC interactors is an important next step in understanding the composition and maintenance of this parasite-host interaction. The interactors could be responsible for generating the electron dense structures observed by electron microscopy. Interestingly VDAC also binds non-polymeric dimeric tubulin (Rostovtseva *et al*., 2008), the binding of which suppresses mitochondrial membrane potential/function. Increased levels of dimeric tubulin could work to the advantage of the parasite by suppressing metabolic activities in non-bound mitochondria explaining why the membrane potential of the mitochondria in infected cells appears suppressed using the JC-1 dye.

### Conclusions

In this study we show that the microsporidium *E. cuniculi* facilitates a fascinating re-orchestration of its host cell environment by inducing the targeted binding of mitochondria to the early vegetative cell stages which are dependent on the ATP supply of the host. Not only does *E. cuniculi* benefit from the spatial proximity of the mitochondria which form a layer around the PVM but the organization of the PV itself is also structured in a way that ensures a maximum of membrane surface being available for this interaction of the growing vegetative cell stages which are exclusively found in the periphery of the PV. These observations correlate well with the shift in energy requirements observed in other microsporidians (Heinz *et al*., 2012), as the differentiating cell stages (sporonts, sporoblasts and eventually spores) progressively lose contact with the PV membrane and become more independent of host cell mitochondrial metabolism. By using rigorous quantitative electron microscopy we were able to show that *E. cuniculi* not only influences the distribution of host cell organelles but also has impact on the distribution of ATP-gating channels in the mitochondrial outer membrane which has not been published before.

Thus overall this remarkable form of host–parasite interaction can be summarized as a combination of (1) optimizing the localization of the ATP-dependent parasite cells in relation to the cytoplasm, (2) a targeted and direct binding of mitochondria to those cell stages at the vacuole–cytoplasm interface and (3) the active re-distribution and concentration of the ATP-delivery system in the mitochondria that are bound to the vacuole membrane to maximize the supply of ATP from the host.

Future work will focus on analysing the nature and the molecular mechanisms involved in this parasite-induced organelle binding and the identification of participating host and parasite genes, with an emphasis on how microsporidians accomplish such a profound interaction with such a tiny genome.

## Experimental procedures

### Cells and parasite culture

The microsporidians *E. cuniculi* and *T. hominis* were individually cultured in monolayer RK-13 cells at 35°C in a 5% CO_2_ incubator in Minimal Essential Medium (MEM) containing GlutaMAX™ supplemented with 10% heat-inactivated fetal calf serum, Kanamycin 100 μg ml^−1^, Penicillin 100 μg ml^−1^, Streptomycin 100 μg ml^−1^ and Fungizone 1 μg ml^−1^.

### Electron microscopy

Monolayer RK-13 cells infected with either *E. cuniculi* or *T. hominis* were grown to near confluency and fixed in 0.5% glutaraldehyde in 0.2 M PIPES buffer (pH 7.2) for 15 min and then scraped from the culture flask in a small volume of the fixative. The cells were then pelleted (15 min at 16.000* g*) and washed three times in 0.1 M sodium cacodylate (pH 7.2) followed by post-fixation for 1.5 h in 1% osmium tetroxide in 0.1 M sodium cacodylate. For some experiments the osmium contained 1.5% w/v potassium ferrocyanide to improve membrane contrast. To accentuate cytoplasmic fibrillar components in broken cells (see below), the osmium step was followed by either (a) 1% w/v tannic acid in 0.05 M sodium cacodylate followed by 5 min incubation in 1% sodium sulfate in the same buffer or (b) ‘*en bloc*’ staining with 3% w/v uranyl acetate overnight. All samples were subsequently washed and dehydrated through a graded ethanol series and embedded in epoxy resin. Ultrathin sections (70 nm) were collected on pioloform-coated EM copper grids (Agar Scientific, Stansted, UK) and contrasted with lead citrate with or without prior staining in 3% w/v uranyl acetate. Sections were inspected using a JEOL 1200EX transmission electron microscope at 80 kV and images taken on Ditabis imaging plates (DITABIS Digital Biomedical Imaging Systems AG, Pforzheim, Germany) or with a Gatan Orius 200 digital camera (Gatan, Abingdon Oxon). Some micrographs were false-coloured in Photoshop (PS CS6) to highlight the localization and interaction of mitochondria and PV in infected cells.

The spatial relationship between mitochondria and PV membrane was also investigated using electron tomography. Epoxy resin sections were cut at a nominal thickness of 250 nm and mounted on EM grids. Ten-nanometre protein A-gold was applied to the grids before and after mounting the sections to provide fiducial markers. The sections were contrasted with 3% w/v uranyl acetate and lead citrate and tilt series recorded on a JEOL JEM-2200FS microscope with energy filter operated at 200 kV. Specimens were tilted along one axis through 120°, from +60° to −60°. The tilt series were analysed and processed using the IMOD software bundle (Kremer *et al*., 1996). Electron tomograms were generated by fiducial alignment in the tilt series and videos reconstituted from consecutive snapshots with ImageJ (Rasband, 1997–2013).

For immuno-gold labelling of infected RK cells samples were also processed according to the Tokuyasu method for cryofixation and ultrathin sectioning (Tokuyasu, 1973). Aldehyde-fixed cells (4% paraformaldehyde, 0.05% glutaraldehyde in 0.2 M PIPES, pH 7.2) were washed three times with buffer and cryoprotected in 2.3 M sucrose in PBS overnight at 4°C. Small fragments of the cell pellet were then plunge-frozen in liquid nitrogen and 80-nm-thick sections were cut at −100°C (EM FC7 ultracryomicrotome; Leica, Vienna, Austria) and stored at 4°C on pioloform-coated EM copper grids (Agar Scientific, Stansted, UK) in drops of 1:1 pre-mixed 2.1 M sucrose/2% w/v methyl cellulose. Prior to labelling, grids were washed in ice-cold distilled water (×3) followed by PBS at room temperature. The sections were then incubated in 0.5% fish skin gelatin (Sigma Aldrich, Poole, UK) in PBS, and labelled using polyclonal rabbit antibody against VDAC (ab96140 raised against peptide from within residues 150–250 of human VDAC; Abcam, Cambridge, UK) followed by 10 nm protein-A gold and contrasted using 2% w/v methylcellulose/3% w/v uranyl acetate. For quantification, labelled sections were analysed in five individual experiments by taking 25–53 micrographs including the detected regions of the parasitophorous vacuole membrane at a nominal magnification of 5000×. Tiff files of micrographs were further analysed using Adobe Photoshop CS6. Randomly placed square lattice grids were placed on each micrograph and used to estimate the membrane boundary lengths of the organelles of interest by intersection counting (grid spacing 373 nm for mitochondria; 932 nm for endoplasmic reticulum, plasma membrane and Golgi apparatus which yielded a total of 86 to 206 intersection counts per quantified mitochondrial membrane compartment). For the assessment of the gold particle localization at the mitochondrial-PV membrane interaction site gold particles were categorized according to their location in respect to the mitochondrial membranes (gold particle either at the outer aspect of the outer mitochondrial membrane, in the intermembrane space between outer and inner mitochondrial membrane or at the inner aspect of the inner mitochondrial membrane) and compared to the localization of the gold particles in mitochondria not bound to the parasite vacuole (101 gold particles for bound mitochondria, 90 gold particles for free mitochondria).

### Broken cells and proteases assays

To investigate the nature and stability of the mitochondrial attachment to the PV membrane cells were broken mechanically and treated with purified proteases. RK-13 cells infected with *E. cuniculi* were grown to near confluency in a 75 cm^2^ culture flask and washed four times in pre-cooled KSHM buffer (100 mM KCl, 85 mM sucrose, 1 mM magnesium acetate and 20 mM HEPES-NaOH, pH 7.4) on ice. The cells were then scraped into 1 ml of KSHM containing proteases (proteinase K, α-chymotrypsin or subtilopeptidase A; Sigma, Dorset, UK) added to 0.1 mg ml^−1^ from 10 mg ml^−1^ stock solutions (prepared in KSHM), and incubated for 1 h on ice. As a control, an aliquot of broken cells in an equal volume of KSHM buffer was incubated in parallel. Reactions were stopped by adding an equal volume of 1:1 KSHM/2% glutaraldehyde in 0.4 M sodium cacodylate and fixed for 15 min on ice followed by pelleting at 7.000 *g* at 4°C for 10 min. After fixation for a further 30 min at room temperature, the pellets were washed three times with 0.1 M sodium cacodylate and processed for epoxy resin-embedding as described above.

To assess the proportion of PV covered mitochondria, approximately 20 micrographs were recorded with a Gatan Orius 200 camera at a nominal magnification of 1200× and then analysed using line intersection counting in Adobe Photoshop CS6 (see below). A cell was defined as broken if there was drastic reduction in contrast and granularity of the cytosol associated with swelling of mitochondria or endoplasmic reticulum.

### Spatial stereology

The volume density of mitochondria around the PV was estimated at various distances from the PV membrane by randomly placing a systematic grid lattice (point spacing 183 nm) over micrographs (*n* = 21) containing profiles of PVs and point counting. The distance from each point in the host cell cytosol to the nearest portion of PV membrane was measured and the points classified as either ‘mitochondria’ (M) or ‘not mitochondria’ (NM) depending on whether they lay on a mitochondrion or a different organelle or an empty portion of cytosol. Features such as shape, size, and the presence of a double membrane and/or cristae were used to identify mitochondria. The point counts were categorized into eleven 100 nm groups, or layers, depending on their distance from the PV membrane (from layer 1: 0–100 nm to layer 11: > 1 μm) and the volume density of each layer was calculated (about 100–200 points for each band of 100 nm around the PV). Depending on what structure was adjacent to the PV membrane the point was classified as either ‘spore’, ‘meront’ or ‘PV membrane’ (PVM). Spores were defined by the presence of a circular or ovoid profile surrounded by a spore wall, which was often visible as a thick layer surrounding the cell. Meronts lacked a curvilinear plasma membrane surrounded discernible cell wall and contained abundant endoplasmic reticulum. Locations on the PV membrane were classified as ‘PV membrane’ when there was neither meront nor spore adjacent to this part of the PV membrane.

To estimate the fraction of the total PV membrane surface in direct contact with mitochondria line intersection counting with square lattice grids was used (point spacing 2.25 μm for untreated cells and 1.87 μm for experiments with broken cells). In untreated cells intersections were classified as either ‘spore’, ‘meront’ or ‘PV membrane’ depending on what structure was adjacent to the PV membrane and categorized by the underlying organelles resulting in 12 groups of data per experiment. This was not possible in broken cells because of the modified morphology. As a control, the fraction of plasma membrane and nuclear envelope surface occupied by mitochondria was also estimated using intersection counting.

The total membrane surface of mitochondrial profiles that were attached to the PVM was estimated by intersection counting on micrographs taken of consecutive serial sections by randomly placing a systematic grid lattice (grid spacing 347 nm) on micrographs of the series and tracing complete mitochondrial profiles bound to the PVM (*n* = 10 mitochondrial profiles present in 3 to 18 serial sections giving a yield of 488 intersection counts in total). The intersection counts were classified as either ‘bound’ or ‘unbound’ membrane fraction of the mitochondrial profile to the PVM.

To estimate the mean distance of both meronts and spores to the nearest mitochondrial profile we measured this distance between the plasma membrane of meronts and the cell wall of spores to the closest mitochondrial profile on the same micrographs used for intersection counts stated in the paragraph above. Forty profiles of both meronts as well as spores were used in the analysis.

### Mitochondrial activity assay

*Encephalitozoon cuniculi*-infected RK-13 cells were grown in a six-well plate near confluency and incubated with the fluorescent dye JC-1 (Invitrogen, Paisley, UK) at a final concentration of 5 μM in culture medium for 30 min at 37°C. JC-1 accumulates in mitochondria in a potential-dependent manner and emits fluorescence in the red spectrum (590 nm) indicating functionally active mitochondria (Smiley *et al*., 1991). After three washes with fresh medium the living cells were immediately imaged using an inverted fluorescent microscope (Axiovert, Zeiss, Cambridge) at 40× magnification. In two individual experiments images of PVs were taken systematic uniform random (SUR, Lucocq, 2012). For quantification of the fluorescent intensity of JC-1 *E. cuniculi*-infected RK-13 cells were sampled in a quadrat (0.031 mm^2^; *n* = 31 cells) placed centrally on the images which were displayed in Adobe Photoshop CS6. The mean fluorescent intensity of the red emitting channel was measured in small quadrats (0.97 μm^2^) in the cytoplasm directly adjacent to the PV or nucleus. The quadrats were placed at the left edge of intersections of the PV or nuclear membrane with a randomly placed grid lattice (grid spacing 22.12 μm, *n* = 2 to 5 measurements per nucleus or PV). As a control the intensity of JC-1 in uninfected cells was measured adjacent to the nucleus in quadrats as described above. Uninfected cells were sampled using the forbidden line unbiased counting rule applied to quadrats positioned SUR (4 quadrats per micrograph, 0.0019 mm^2^ per quadrat; *n* = 83 cells) on the same set of images. Non-cellular background fluorescence was evaluated at three to six positions closest to the cells that had been assessed and the mean fluorescent intensity subtracted from the recorded JC-1 intensities for each image. The area of every PV was estimated by point counting using a randomly placed square grid lattice in Photoshop (grid spacing 4.42 μm, which yielded a total of 101 to 254 points per experiment). To estimate the local density of mitochondria at locations used for JC-1 analysis we estimated the volume density of mitochondria using point counting on a randomly placed grid lattice (grid spacing 374 nm, which yielded 105 to 108 points per compartment area) on 17 electron micrographs. In each case quadrats enclosing the same area and aligned applying the same criteria as used for the JC-1 fluorescence imaging were used.

## Supporting Information

**Fig. S1.** Membrane distortions at the PVM–OMM interface. At irregular intervals the interaction site of mitochondria attached to the PVM appeared distorted with deformations extending from the OMM to the plasma membrane of *E. cuniculi* indicative of active binding rather than mere clustering.

A. Mitochondrial profile in close contact with the PVM of a meront cell stage. Numerous membrane distortions can be seen between the meront plasma membrane and the OMM (see black arrows).

B. A similar membrane deformation visible in a region of mitochondrial contact with a spore. The distortion extends over the thick cell wall of the spore (see arrows). CW, cell wall; M, mitochondrion; Me, meront; N, nucleus; Sp, spore; PM, plasma membrane; PVM, parasitophorous vacuole membrane.

**Fig. S2.** Fraction of meront and spore membrane in contact with PVM. The mean fraction of the meront plasma membrane in contact with the PVM and surrounded by host cell cytoplasm was 65.78%, indicating that meronts bulge out into the cytoplasm. Conversely, the fraction of spores abutting the cytosol at the PVM interface was much lower (27.29%). Error bars indicate the SEM.

**Fig. S3.** Electron tomogram of a 250 nm thick section of *E. cuniculi* meront in contact with several host mitochondria. The tomogram (see *Experimental procedures*) reveals the interaction between the attached mitochondria and the PVM, which appears seamless over the full length of each mitochondrial profile. There is a consistent intermembrane spacing between outer mitochondrial membrane and PVM. Electron dense linkages between both membranes can be seen at various locations throughout the tomogram.

**Fig. S4.** Association of endoplasmic reticulum with the *E. cuniculi* PV. The ER cisterna closely follows the curvature of the PVM but does not seem to be in continuous contact throughout its profile length. Both the PVM and the ER membrane appear corrugated along the length of the contact site. A total of 8% of the PVM surface displayed such an ER attachment. Inside the PV lumen two profiles of spore stages can be seen.

**Fig. S5.** Host organelle localization in RK-13-cells infected with the microsporidium *T. hominis.*Mitochondria do not show any apparent attachment around the PV of *T. hominis*.

A. Fraction of the PVM membrane surface in regions of PVM overlaying either meront or spore stages of *T. hominis* in contact with mitochondria.

B. Mitochondria false-coloured in orange; PVM false-coloured in green.

**Fig. S6.** Distribution of length measurements of OMM-PVM intermembrane space and electron dense linkages. The distance between the OMM and the PVM was measured at mitochondrial profiles bound to the PV (see *Experimental procedures*). The distances were then plotted as a frequency distribution as shown. This highlights a marked clustering of measurements between 2.2 and 8.2 nm.
