# Supplementary figures and images for: Strategies for maximizing ATP supply in the microsporidian Encephalitozoon cuniculi: direct binding of mitochondria to the parasitophorous vacuole and clustering of the mitochondrial porin VDAC
Source: Cell Microbiol. 2013 Dec 6;16(4):565–79. doi: 10.1111/cmi.12240 (PMC4233961; doi:10.1111/cmi.12240)

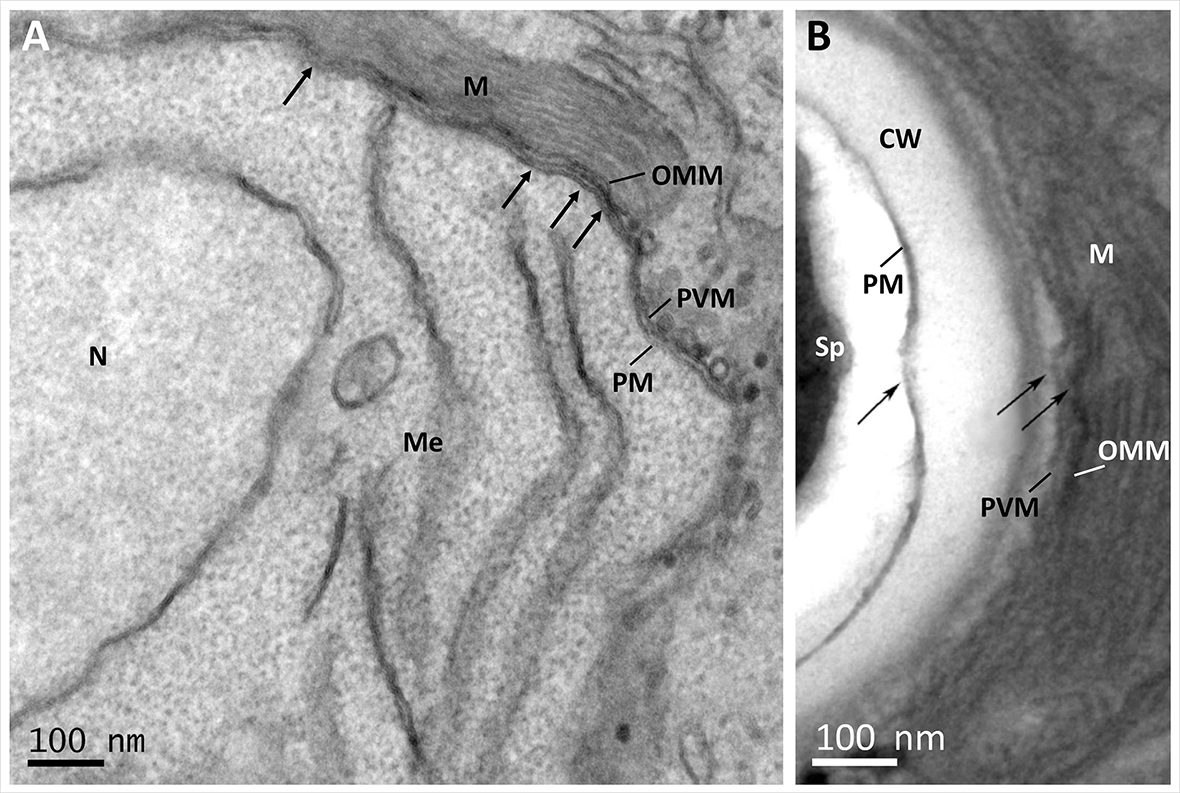

Supplement: Supplementary file 1 — Supplementary [file cmi0016-0565-SD1.tif]

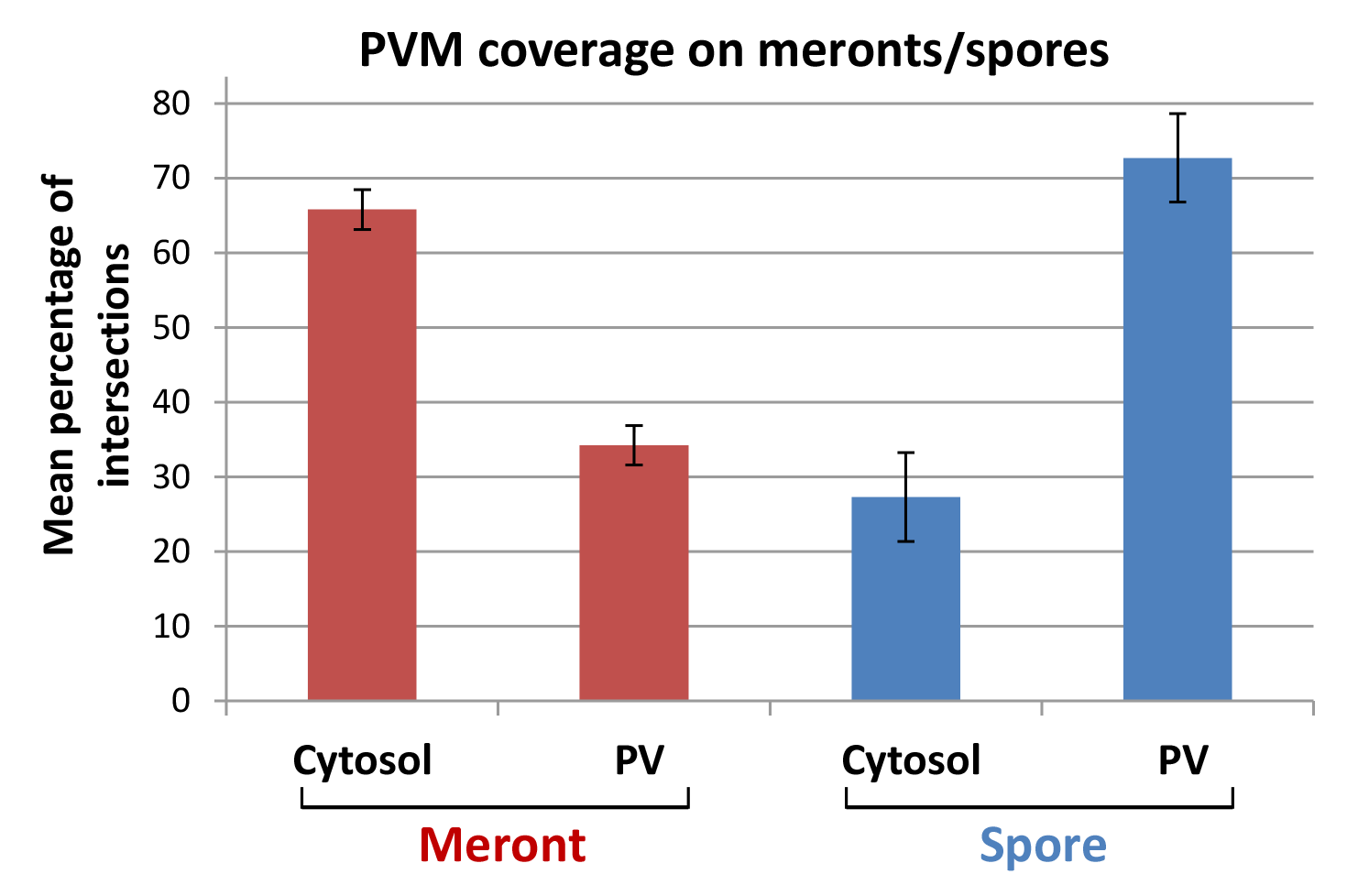

Supplement: Supplementary file 2 — Supplementary [file cmi0016-0565-SD2.tif]

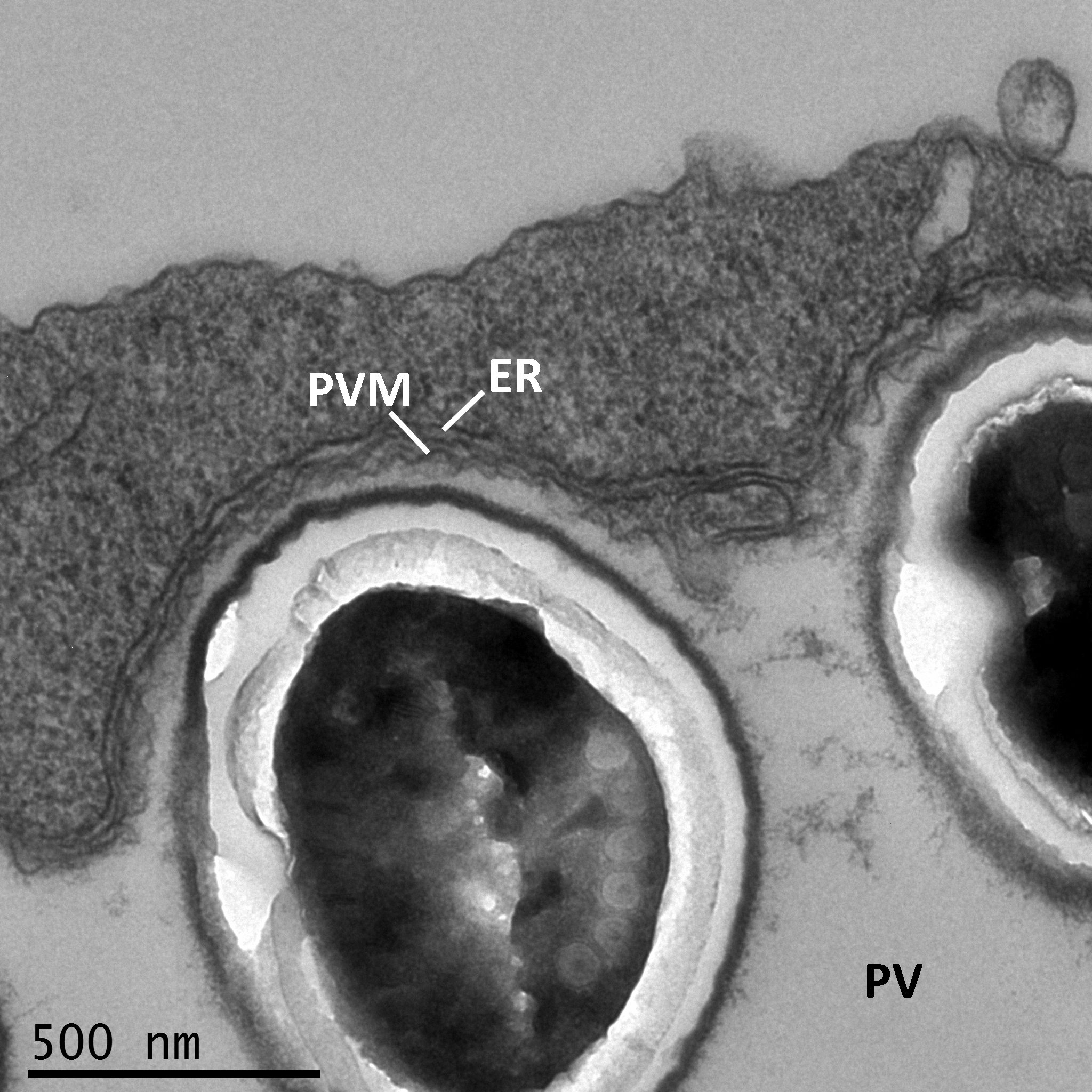

Supplement: Supplementary file 4 — Supplementary [file cmi0016-0565-SD4.tif]

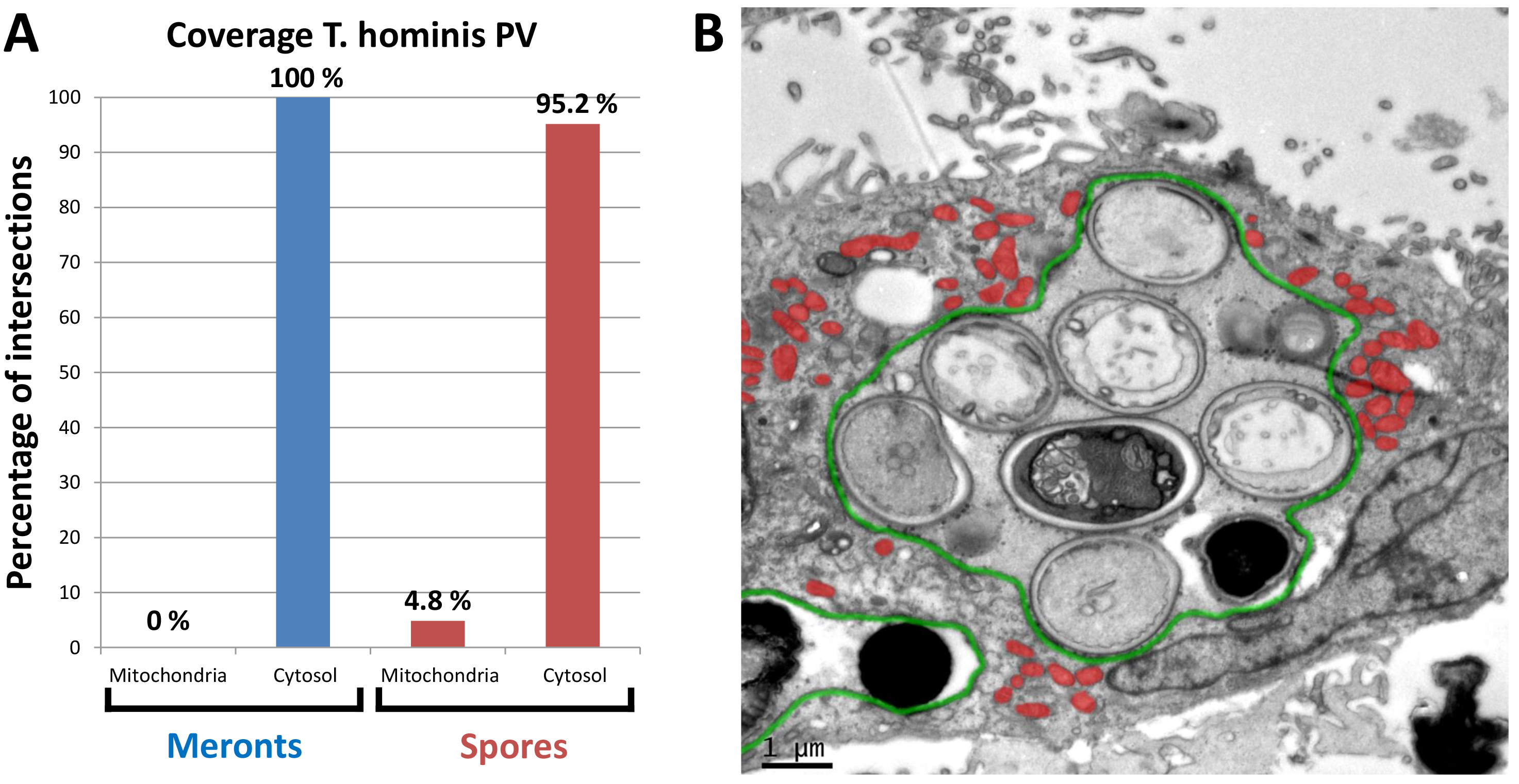

Supplement: Supplementary file 5 — Supplementary [file cmi0016-0565-SD5.tif]

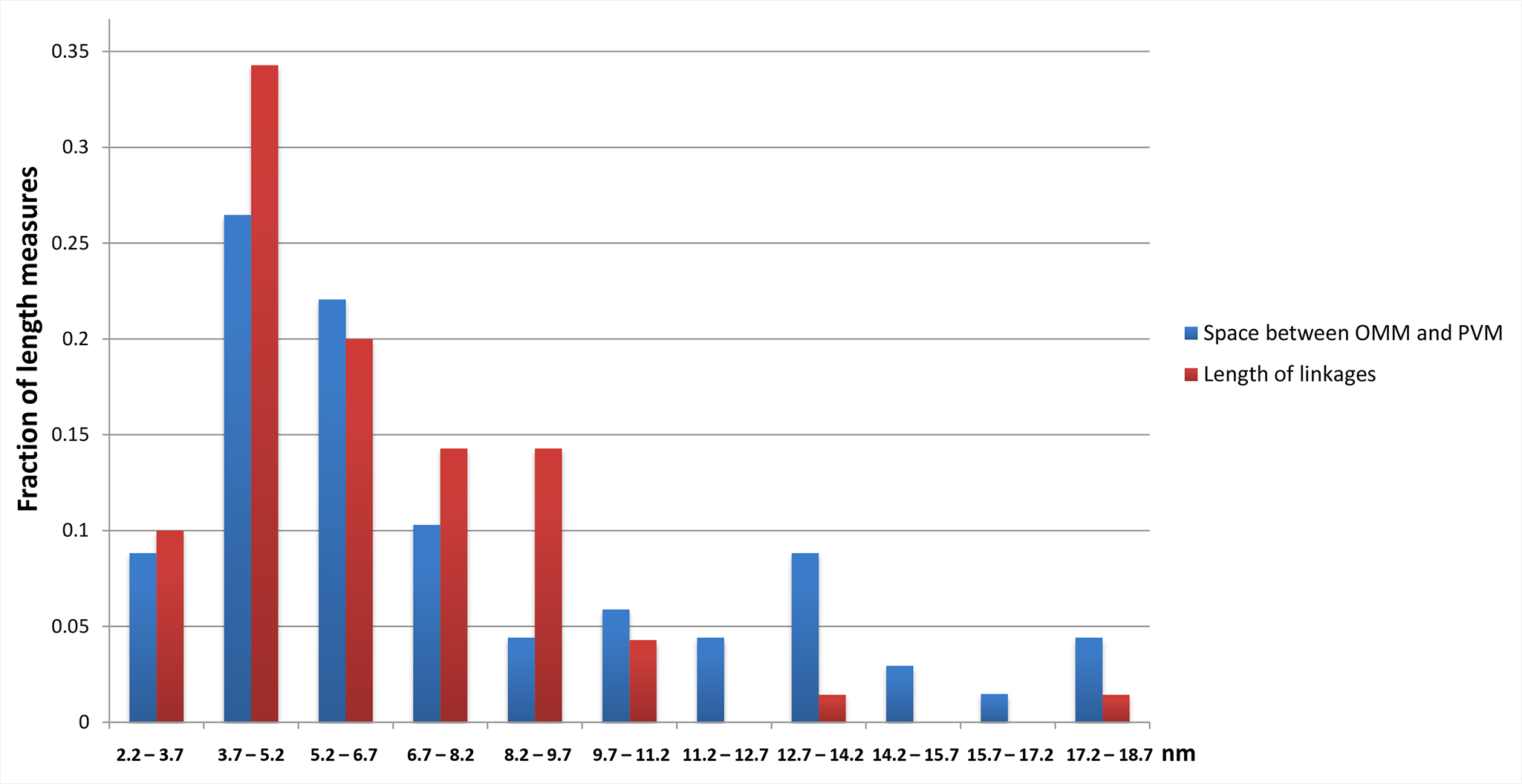

Supplement: Supplementary file 6 — Supplementary [file cmi0016-0565-SD6.tif]
